# Prognostic significance of neutrophil-to-lymphocyte ratio in non-small cell lung cancer: a meta-analysis

**DOI:** 10.1038/srep12493

**Published:** 2015-07-24

**Authors:** Xiao-Bin Gu, Tian Tian, Xiao-Jing Tian, Xiao-Jun Zhang

**Affiliations:** 1 Cancer Center, Chinese PLA General Hospital and Chinese PLA Medical School, Beijing, China; 2 Nanlou Department of Respiratory Disease, Chinese PLA General Hospital and Chinese PLA Medical School, Beijing, China

## Abstract

Published data on the prognostic significance of neutrophil-to-lymphocyte ratio (NLR) in non-small cell lung cancer (NSCLC) are controversial. We performed a meta-analysis to more accurately assess its prognostic value. The analysis was performed based on the data from 14 studies with 3,656 patients to estimate the correlation between NLR and overall survival (OS) and progression-free survival (PFS) in NSCLC. Hazard ratio (HR) with 95% confidence interval (CI) were calculated to estimate the effect. We also conducted subgroup analysis and meta-regression analysis. The results demonstrated that elevated pretreatment NLR predicted poorer OS (HR: 1.70, 95% CI: 1.39–2.09) and PFS (HR: 1.63, 95% CI: 1.27–2.09) in patients with NSCLC. Subgroup analysis indicated that cut-off value of 5 showed consistently prognostic value. There was no significant heterogeneity or publication bias for OS and PFS for included studies. This meta-analysis revealed that elevated pretreatment NLR might be a predicative factor of poor prognosis for NSCLC patients.

Lung cancer is one of the most commonly diagnosed cancers and remains the leading cause of cancer-related death worldwide^1^. Non-small-cell lung cancer (NSCLC) accounts for approximately 80–85% of all lung cancer cases. Despite diverse treatment methods including surgery, chemotherapy, radiation and targeted therapies are used, the prognosis of NSCLC is disappointing, with 5-year survival rate remains about 17%^2^. The high mortality rates of NSCLC are partly due to the lack of effective prognostic biomarkers. Therefore, it is urgent for us to identify novel prognostic factors which might enable clinicians to stratify risk patients and further tailor therapeutic strategies.

Up to now, a series of traditional prognostic parameters for NSCLC patients are well known. Several independent prognostic factors for survival in patients with NSCLC have been identified: age, sex, weight loss, smoking status, performance status and TNM stage^3^. However, these factors are not adequately used in clinical settings for insufficient specificity and sensitivity. In recent years, accumulating evidence demonstrated that systemic inflammatory response is associated with poor prognosis in various solid tumors^4^,^5^,^6^,^7^. Distinct index or markers of systemic inflammatory response such as Glasgow Prognostic Score(GPS), C-reactive protein (CRP) and neutrophil-to-lymphocyte ratio(NLR) have been evaluated in a series of cancers^8^,^9^. These studies demonstrate that tumor cells can recruit neutrophils into the tumor stroma through specific chemokines^10^. Subsequently, neutrophils exert pro-tumorigenesis effects by inhibiting apoptosis, promoting angiogenesis and metastasis^11^,^12^. While, infiltrating lymphocytes which play a role in tumor defence are associated with favorable prognosis^13^. So the NLR, which is defined as neutrophil counts divided by lymphocyte counts, is particularly noteworthy.

Gathering evidences have indicated that NLR had prognostic significance in patients with breast cancer, colorectal cancer, renal cell carcinoma, gastric cancer and hepatocellular carcinoma^14^,^15^,^16^,^17^,^18^. Recent studies suggest a potential prognostic role of NLR in NSCLC patients, however, the majority of the studies had relatively limited sample sizes^19^,^20^,^21^,^22^,^23^. Furthermore, some authors presented conflicting data regarding the prognostic significance of NLR in NSCLC^24^. We thus conducted this meta-analysis to systematically clarify the prognostic value of NLR in NSCLC patients.

## Results

### Study selection and characteristics

**Figure 1:**
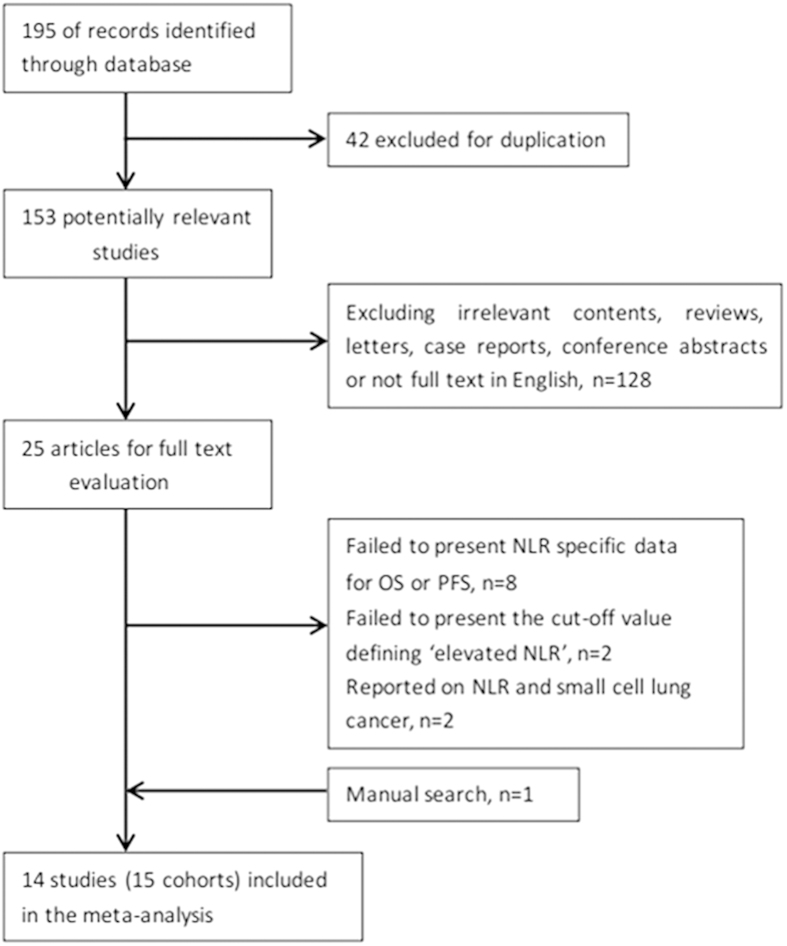
Flow chart of the included studies.

The flow chart of the literature selection was shown in Fig. 1. The initial search strategies retrieved a total of 195 studies. After screening the titles or abstracts, 170 studies were excluded as they were either duplicate reports, conference abstracts, reviews, case reports, reports in language other than English or studies irrelevant to the current analysis. Then, 25 identified studies concerning NLR and the prognosis of NSCLC were further evaluated. Eleven reports of them were discarded because of the following reasons: eight did not provide specific NLR data for OS or PFS, two failed to define cut-off value of “elevated NLR”, two reported on NLR and small cell lung cancer, we also added one article by manual search. Therefore, 14 studies^19^,^20^,^21^,^22^,^23^,^24^,^25^,^26^,^27^,^28^,^29^,^30^,^31^,^32^ with 3656 patients published between 2009 and 2015 were included in our meta-analysis finally. As the study by Botta *et al.*^28^ included two cohorts and reported the HR and 95%CI respectively, we marked them as Botta1 and Botta2. The main characteristics of these studies are shown in Table 1. Three studies were conducted in USA^21^,^30^,^32^, two studies were performed in Japan^25^,^26^, China^20^,^31^ and Turkey^19^,^22^, respectively, one in Spain^27^, Italy^28^, Korea^24^, Belgium^29^, and UK^23^, respectively. One study^22^ involved all disease stages, six studies^19^,^21^,^23^,^26^,^29^,^32^ included only early stage disease (І/І-ІІ/І-ІІІ/ІІ-ІІІB) and seven studies^20^,^24^,^25^,^27^,^28^,^30^,^31^ included only late stage disease (ІІІB-ІV/ІV).Thirteen studies^19^,^20^,^21^,^22^,^23^,^24^,^25^,^26^,^27^,^29^,^30^,^31^,^32^ with 3,544 patients reported the correlations of NLR and OS, while nine studies^20^,^21^,^24^,^25^,^28^,^29^,^30^,^31^,^32^ (ten cohorts) with 2,623 patients reported the correlations of NLR and PFS. NOS scores of the studies ranged from 5 to 8, with a mean value of 6.64.

### NLR and OS in NSCLC

**Figure 2:**
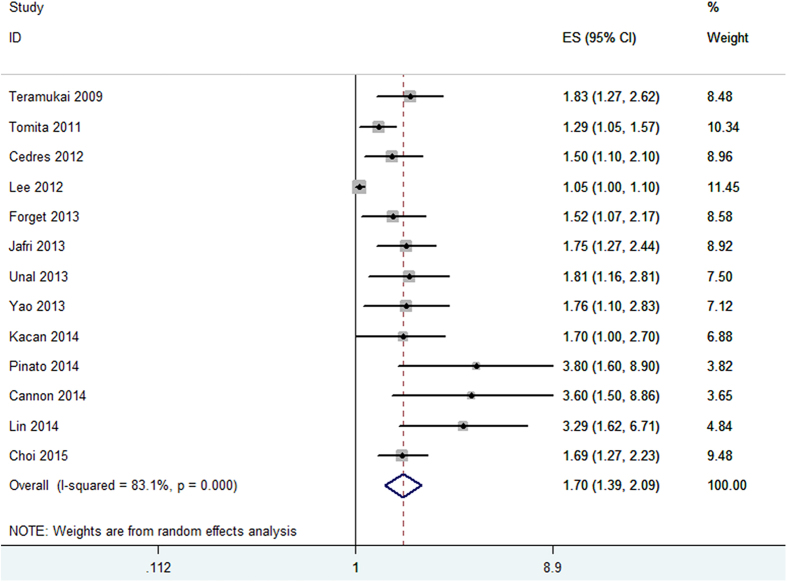
Forrest plots of studies evaluating hazard ratio (HR) with 95% CI of NLR for overall survival(OS).

Thirteen cohorts presented the data of pretreatment NLR and OS in NSCLC patients. Meta-analysis of these 13 cohorts showed that patients with elevated NLR were associated with shorter OS (HR obtained from DerSimonian–Laird random-effects model: 1.70 (95% CI: 1.39–2.09, p < 0.001); Fig. 2), although there was heterogeneity between studies (I^2^ = 83.1%, Ph < 0.001). Then we conducted subgroup analyses according to confounders such as treatment method, study location, tumor stage, sample size, cut-off value defining “elevated NLR” and NOS score.

Stratification by treatment methods, we found the pooled HRs were 1.70 (95%CI: 1.39–2.10) for patients treated by surgery and 1.76 (95%CI: 1.30–2.39) for patients treated by non-surgery methods. Subgroup analyses by countries indicated that elevated NLR predicted poor prognosis for patients both in western countries (HR = 1.74, 95%CI: 1.44–2.12) and in eastern countries(HR = 1.58, 95%CI: 1.22–2.04). Stratification by cutoff value = 5 and cut-off value ≠ 5, the data showed that the pooled HR was 1.67 (95%CI:1.44–1.94) for cutoff value = 5 and 1.67 (95%CI:1.26–2.23) for cut-off value ≠ 5. Notably, when cut-off value = 5 was used, there was no heterogeneity (I^2^ = 0, Ph = 0.506), which may indicate NLR = 5 is more stable in prognosis prediction. In addition, subgroup analyses showed the elevated NLR predicted prognosis for NSCLC regardless of tumor stage (early stage vs. late stage), sample size(≥200 vs.<200) and NOS score(≥7 vs.<7) (Table 2).

### NLR and PFS in NSCLC

**Figure 3:**
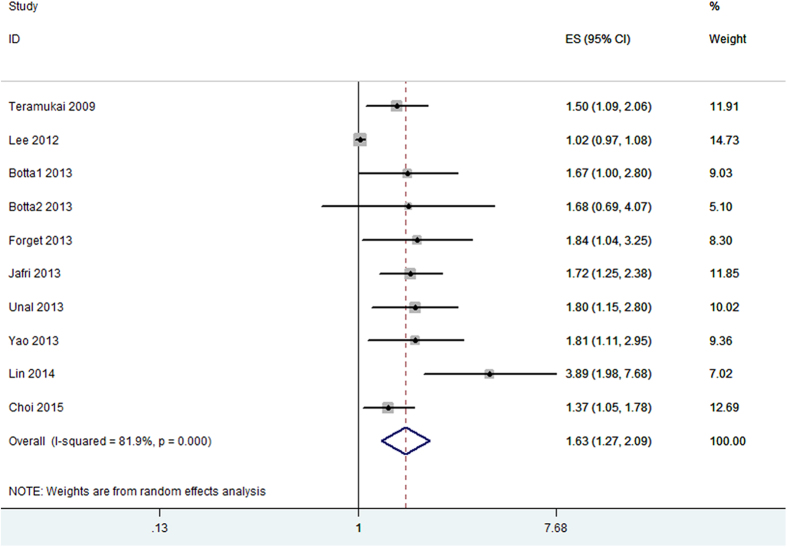
Forrest plots of studies evaluating hazard ratio (HR) with 95% CI of NLR for progression-free survival(PFS).

Ten cohorts with 2,623 cases reported the data of pretreatment NLR and PFS in NSCLC patients. Combined data from the ten cohorts suggested that elevated pretreatment NLR were significantly correlated with PFS with a pooled HR estimate of 1.63 (95% CI: 1.27–2.09, p < 0.001; Fig. 3), with heterogeneity (I^2^ = 81.9%, Ph < 0.001). Subgroup analysis indicated that elevated pretreatment NLR were significantly associated with PFS in weastern countries (HR: 1.56, 95% CI: 1.31–1.86, p < 0.001), without significant heterogeneity in the data (I^2^ = 0, Ph = 0.791). We did not perform subgroup analysis for PFS based on treatment method as majority therapeutic regimen in the studies was chemotherapy. Moreover, elevated pretreatment NLR was also associated significantly with PFS in NSCLC patients with a cut-off value of 5 (HR: 1.54, 95% CI: 1.27–1.86, p < 0.001), without significant heterogeneity in the data (I^2^ = 0, Ph = 0.453).

### Heterogeneity

**Figure 4:**
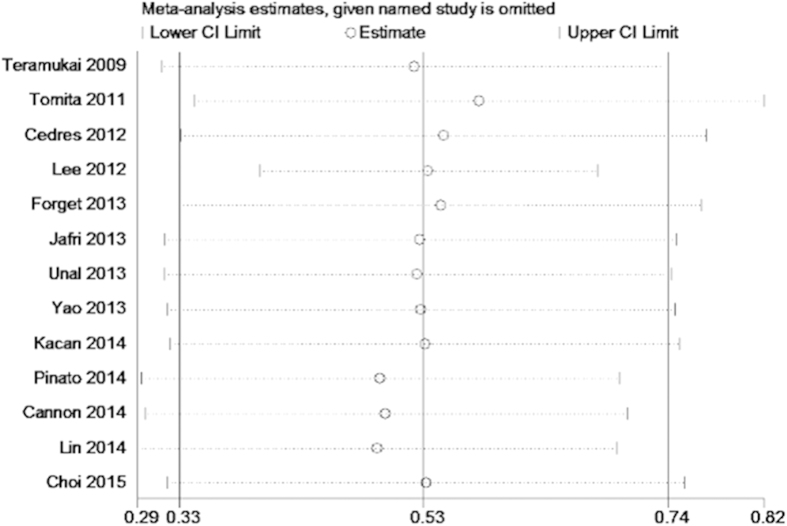
Sensitivity analysis on the relationship between NLR and OS in NSCLC.

**Figure 5:**
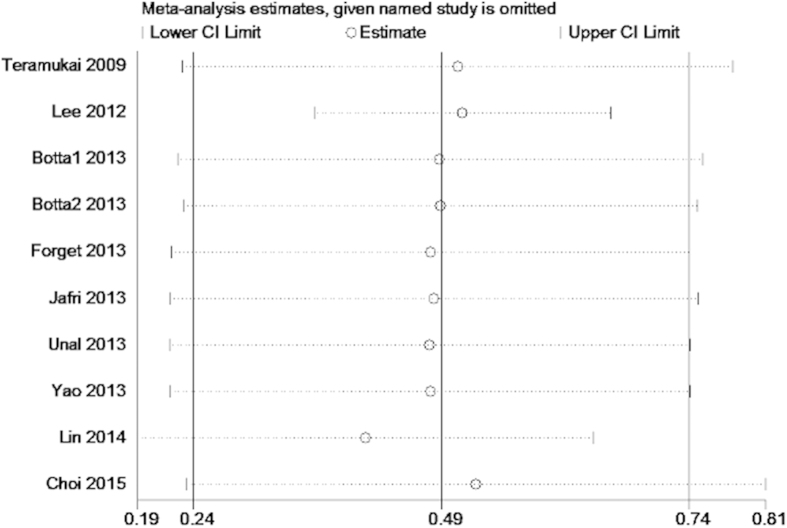
Sensitivity analysis on the relationship between NLR and PFS in NSCLC.

We conducted meta-regression analysis to investigate the potential source of heterogeneity among studies for OS and PFS. The results showed that treatment method (p = 0.891), study location(p = 0.387), tumor stage(p = 0.625), sample size(p = 0.97), cut-off value (p = 0.693) and NOS score (p = 0.084) did not contribute to the source of heterogeneity for OS. Moreover, the data demonstrated that study location(p = 0.944), sample size(p = 0.733) and NOS score (p = 0.202) did not contribute to the source of heterogeneity for PFS. Sensitivity analysis indicated that removing any single study by turn did not significantly affect the pooled HRs for OS and PFS (Figs 4 and 5).

### Publication bias

Publication bias estimate was mainly used to evaluate the reliability of meta-analysis results, especially which showed statistical significance^33^. Assessment of publication bias by using Begg’s test (statistical significance was set at p < 0.05) suggested that were no significant publication bias in OS and PFS studies (p = 0.2 and p = 0.721, respectively).

## Discussion

This meta-analysis aimed to examine the associations between elevated pretreatment NLR and OS and PFS of NSCLC. Our analysis combined the outcomes of 3,656 NSCLC patients from 14 individual studies, demonstrating that elevated pretreatment NLR significantly predicted poor OS (HR: 1.70, 95% CI 1.39–2.09), and PFS (HR: 1.63, 95% CI 1.27–2.09) of NSCLC cancer patients. Although heterogeneity exists, most of the prognostic significance is not weakened by subgroup analysis stratified by treatment method, study location, tumor stage, sample size, cut-off value of NLR and NOS score. Furthermore, subgroup analysis indicated that NLR had consistent prognostic value for NSCLC populations of OS with a cut-off value of 5. Whereas, NLR could better predicted poor PFS for NSCLC patients in western countries with a cut-off value of 5. This finding suggested that dichotomized NLR cut-off value of 5 could help guide clinical decision-making in regard of therapeutic strategies and outcomes for NSCLC patients both for OS and PFS. To the best of our knowledge, this is the first meta-analysis on the association between elevated pretreatment NLR and clinical outcomes in NSCLC.

Accumulating evidence showed the connection between inflammation and cancer and mechanistic studies have presented solid evidence to support the biological and prognostic importance of a pro-inflammatory tumor microenvironment in cancer progression^7^,^34^. An elevated NLR implies an increased neutrophil count and/or a decreased lymphocyte count, as well as a relative lymphopenia. Lymphocytes have an important role in tumor defence, which inhibits tumor cell proliferation and migration^7^,^35^. However, a large amount of neutrophils had been indicated to influence cytolytic activity of lymphocytes or natural killer cells, as well as suppress T-cell proliferation^36^. Thus, neutrophils in the tumor microenvironment could have negative impact on tumor growth. Therefore, NLR could concisely reflect the imbalance of pro-tumor and anti-tumor activity of the hosts in respect of inflammatory response. Thus, the relative value of a combined neutrophil and lymphocyte counts index in form of a neutrophil to lymphocyte (N/L) ratio can reflect the protumor efficacy and antitumor capacity of the host more accurately. IL-17 and peritumoral CD163 may exert important roles in the inflammatory tumor microenvironment and facilitate tumor progression and recurrence^37^. Additionally, it is convenient and cost-effective to measure the parameter of NLR in clinical practice, which makes NLR an attractive biomarker for NSCLC prognostication.

More recently, several meta-analyses reported the prognostic value of NLR in a variety of cancers, including colorectal cancer, hepatocellular carcinoma, gastric cancer, renal cell carcinoma, pancreatic cancer and esophageal cancer^17^,^18^,^38^,^39^,^40^,^41^. Our study was the first study investigating the prognostic significance of NLR for NSCLC patients and the results were in line with previous reports, indicating that elevated NLR gained prognostic values for solid tumors and NLR could be widely used in clinical settings, especially for cancer patients. In addition, the value of NLR was easy to obtain because it is a routine test and more importantly, it does not add extra cost. So NLR is a promising biomarker for clinical use.

In spite of the intrinsic defects associated with meta-analysis, there are a number of other limitations in our study. First, significant heterogeneity was observed in the results due to confounding factors, such as the baseline characteristics of the patients, treatment methods, follow-up period, sample size and cut-off value of NLR. However, subgroup analysis, meta-regression analysis and sensitivity analysis showed that none of the above-mentioned confounders could completely explain the heterogeneity. Thus, we supposed that the heterogeneity could be a result of combined effect of the above-mentioned confounders and the genotypic diversity of lung cancer in these studies. Second, we did not analyze the correlation between the elevated NLR and clinicopathological parameters of patients, such as lymph node metastasis, grade of differentiation and tumor stage, because only two studies reported the relevant information. The data is insufficient to analyze. Third, some primary studies evaluated the prognostic role of NLR in univariate analysis, whereas others used multivariate analysis, which may contribute to some bias when the data were pooled. Forth, most of the original studies showed that high NLR predicted poor prognosis due to positive results tend to be published, although two studies^24^,^28^ gained negative results for PFS, more controversial papers could not be searched.

Despite several limitations, our meta-analysis also had some advantages. First, we got similar results when the data were analyzed neither in random-effects model nor in fixed-effects model, which indicated that robustness of the statistic results. Second, the results of sensitivity analysis did not significantly altered, indicating that our results were stable. At last, all the scores of study quality assessed by NOS were ≥5, which demonstrated the creditability of our meta-analysis results.

In conclusion, our results indicated that elevated pretreatment NLR might be an unfavorable prognostic factor for patients with NSCLC, which could be useful in stratifying patients and in determining individual treatment plans. However, these findings need to be interpreted cautiously when used in clinical practice because of the limitations listed above. More well-designed and large-scale investigations are warranted to better understand the value of NLR in the prognosis of NSCLC.

## Methods

### Publication search

A literature search was conducted via Pubmed, Embase, and Web of Science databases for articles that assessed NLR as a prognostic factor for survival of patients with NSCLC (last search was updated on May 6, 2015). The search strategy used key words such as “neutrophil-to-lymphocyte ratio” , “neutrophil lymphocyte ratio” , “NLR” , “lung cancer” , ‘lung carcinoma’, ‘NSCLC”, “non small cell lung cancer”, “non-small cell lung cancer”, “prognosis”, “prognostic” and “survival”. Article language was restrained to English. The references in the identified articles were also retrieved to find other relevant studies.

### Study selection criteria

Two reviewers (X.B.G. and X.J.T.) reviewed all candidate articles independently. Discrepancies were resolved by discussion. Studies were eligible for inclusion in the meta-analysis if they met the following criteria: (a) patients with NSCLC in the studies were confirmed histopathologically; (b) investigated the association of pre-treatment NLR with overall survival (OS) or progression-free survival (PFS); (c) reported a hazard ratio (HR) and 95% confidence intervals (CIs) or the data sufficient to estimate the HR and 95% CIs; (d) to be published as full texts in the English language. Small-cell lung cancer was not included in our study because it is a highly undifferentiated cancer with distinct biological behaviors from NSCLC.

### Data extraction and quality assessment

Two investigators (X.B.G. and T.T.) reviewed each eligible study and extracted data. The extracted data including: first author’s name, study location, publication year, duration of the studies, follow-up period, sample size, tumor stage, predominant treatment methods, study design, cut-off value of “elevated NLR” and HRs with 95% CIs. If not available, data were extracted to calculate HR by the method of Tierney *et al.*^42^. Quality assessment was independently conducted in all the included studies by three investigators (X.B.G., X.J.Z. and X.J.T.) using the Newcastle–Ottawa Quality Assessment Scale (NOS). Disagreements were resolved by discussion. The NOS comprised of three parameters of quality: selection (0–4 points), comparability (0–2 points), and outcome assessment (0–3 points). The maximum possible score is 9 points and NOS scores ≥7 are considered as high-quality studies.

### Statistical analysis

We directly obtained hazard ratio (HR) and 95% confidence intervals(95% CI) from each article or estimated these data according to the methods illustrated by Tierney *et al.*^42^. A test of heterogeneity of pooled results was performed using Cochran’s Q test and Higgins I-squared statistic. I^2^ > 50% is considered as a measure of significant heterogeneity. Both random effects (DerSimonian–Laird method) and fixed-effects (Mantel–Haenszel method) models were used to generate the pooled HRs and 95%CIs. Owing to a tendency of possible heterogeneity between primary studies, the random-effects model was chosen because it was usually more conservative. We also investigated reasons for inter-study heterogeneity using subgroup analysis and meta-regression analysis. Sensitivity analyses were conducted to evaluate the stability of the results. Publication bias of literatures was evaluated using Begg’s funnel plot. All statistical tests were two sided and the significance level was set at 5%. All analyses were carried out using STATA 12.0 software (STATA, College Station, TX).

## Additional Information

**How to cite this article**: Gu, X.-B. *et al.* Prognostic significance of neutrophil-to-lymphocyte ratio in non-small cell lung cancer: a meta-analysis. *Sci. Rep.* **5**, 12493; doi: 10.1038/srep12493 (2015).

## Figures and Tables

**Table 1:** Characteristics of included studies.

Study	Year	Country	Duration	Sample size	Follow-up(m) (median/range)	Stage	Treat-ment	Cut-off value	Survival analysis	Study design	NOS
Teramukai^25^	2009	Japan	2001–2005	388	18.9(2.3–57)	IIIB-IV	C	4.74	OS,PFS	P	8
Tomita^26^	2011	Japan	2000–2005	284	>60	I-III	S	2.5	OS	R	8
Cedres^27^	2012	Spain	2004–2009	171	9.1(1–70.4)	IV	C	5	OS	R	8
Lee^24^	2012	Korea	2005–2007	199	36	IIIB-IV	C	3.17	OS,PFS	P	7
Botta1^28^	2013	Italy	2008–2011	73	15	IIIB-IV	C+T	4	PFS	R	7
Botta2^28^	2013	Italy	2008–2011	39	15	IIIB-IV	C	4	PFS	R	7
Forget^29^	2013	Belgium	1993–2004	255	56.1	I-II	S	5	OS,PFS	R	8
Jafri^30^	2013	USA	2000–2011	173	NR	IV	C	5	OS,PFS	R	6
Unal^19^	2013	Turkey	NR	94	NR	II-IIIB	C	3.44	OS,PFS	R	5
Yao^20^	2013	China	2007–2010	182	NR	IIIB-IV	C	2.63	OS,PFS	R	6
Kacan^22^	2014	Turkey	NR	299	NR	I-IV	S	5	OS	R	5
Pinato^23^	2014	UK	2004–2011	220	12	I-III	S	5	OS	P	7
Cannon^21^	2014	USA	2006–2012	59	17	I	R	2.98	OS	R	6
Lin^31^	2014	China	2009–2012	81	13–40	IV	T	3.5	OS,PFS	R	6
Choi^32^	2015	USA	2004–2010	1139	NR	I-III	S	5	OS,PFS	R	6

NR: not reported; Treatment describes whether the patients received surgery (S), chemotherapy (C), radiotherapy (R) or targeted therapy (T); OS: overall survival; PFS: progression-free survival; Study design describes the studies as either prospective (P) or retrospective (R) study.

**Table 2:** Summary of the meta analysis results.

Outcome	Stratified analysis	No. of studies	No. of patients	Random-effects model		Fixed-effects model		Heterogeneity	
				HR(95%CI)	P	HR(95%CI)	p	I^2^(%)	Ph
OS	Treatment								
	Surgery	5	2197	1.70(1.39–2.10)	<0.001	1.10(1.05–1.15)	<0.001	85.2	<0.001
	Non-surgery	8	1347	1.76(1.30–2.39)	<0.001	1.49(1.30–2.72)	<0.001	46.8	0.111
	Country								
	Western	6	2017	1.74(1.44–2.12)	0.001	1.70(1.46–1.99)	<0.001	82.4	<0.001
	Eastern	7	1527	1.58(1.22–2.04)	<0.001	1.09(1.04–1.14)	<0.001	29.7	0.212
	Tumor stage								
	Early stage	7	2350	1.69(1.37–2.10)	<0.001	1.55(1.36–1.77)	<0.001	48.8	0.068
	Late stage	6	1194	1.64(1.19–2.27)	0.003	1.09(1.04–1.14)	<0.001	85.8	<0.001
	Sample size								
	≥200	6	2585	1.61(1.33–1.95)	<0.001	1.53(1.34–1.75)	<0.001	41.7	0.127
	<200	7	959	1.76(1.26–2.44)	0.001	1.09(1.04–1.14)	<0.001	84.8	<0.001
	Cut–off value								
	=5	6	2257	1.67(1.44–1.94)	<0.001	1.67(1.44–1.94)	<0.001	0	0.506
	≠5	7	1287	1.67(1.26–2.23)	<0.001	1.09(1.04–1.14)	<0.001	84.2	<0.001
	NOS score								
	≥7	6	1517	1.46(1.14–1.86)	0.002	1.09(1.04–1.14)	<0.001	82.2	<0.001
	<7	7	2027	1.83(1.56–2.15)	<0.001	1.83(1.56–2.15)	<0.001	0	0.498
PFS	Country								
	Western	5	1679	1.56(1.31–1.86)	<0.001	1.56(1.31–1.86)	<0.001	0	0.791
	Eastern	5	944	1.68(1.12–2.52)	0.012	1.05(1.00–1.11)	0.049	86.9	<0.001
	Sample size								
	≥200	3	1782	1.46(1.21–1.77)	<0.001	1.46(1.21–1.77)	<0.001	0	0.643
	<200	7	841	1.72(1.20–2.46)	0.003	1.06(1.01–1.12)	0.019	84.5	<0.001
	Cut-off value								
	=5	3	1567	1.54(1.27–1.86)	<0.001	1.54(1.27–1.86)	<0.001	0	0.453
	≠5	7	1056	1.67(1.19–2.35)	0.003	1.06(1.01–1.12)	0.027	82.7	<0.001
	NOS score								
	≥7	5	954	1.40(1.03–1.91)	0.032	1.04(0.99–1.10)	0.115	71	0.008
	<7	5	1669	1.79(1.38–2.33)	<0.001	1.67(1.41–1.98)	<0.001	52.3	0.079

Ph: p value of Q test for heterogeneity test; N: number of studies (cohorts); HR: hazard ratio; 95% CI: 95% confidence interval; For OS and PFS, subgroup analyses were performed by treatment (surgery vs. non-surgery), study location (Western vs. Eastern countries), sample size (≥200 vs.<200) ,cut-off value of NLR (5 vs. not 5) and NOS score(≥7 vs.<7).
